# Getting out and about in older adults: the nature of daily trips and their association with objectively assessed physical activity

**DOI:** 10.1186/1479-5868-8-116

**Published:** 2011-10-21

**Authors:** Mark G Davis, Kenneth R Fox, Melvyn Hillsdon, Jo C Coulson, Debbie J Sharp, Afroditi Stathi, Janice L Thompson

**Affiliations:** 1 Centre for Exercise, Nutrition and Health Sciences, School for Policy Studies University of Bristol, Bristol, UK; 2 School of Sport and Health, University of Exeter, Exeter, UK; 3 Academic Unit of Primary Health Care, School of Social and Community Medicine, University of Bristol, Bristol, UK; 4 School for Health, University of Bath, UK

## Abstract

**Background:**

A key public health objective is increasing health-enhancing physical activity (PA) for older adults (OAs). Daily trip frequency is independently associated with objectively assessed PA volumes (OAs). Little is known about correlates and these trips' transport mode, and how these elements relate to PA. Purpose: to describe the frequency, purpose, and travel mode of daily trips in OAs, and their association with participant characteristics and objectively-assessed PA.

**Methods:**

Participants (n = 214, aged 78.1 SD 5.7 years), completed a seven-day trips log recording daily-trip frequency, purpose and transport mode. Concurrently participants wore an accelerometer which provided mean daily steps (steps·d^-1^), and minutes of moderate to vigorous PA (MVPA·d^-1^). Participants' physical function (PF) was estimated and demographic, height and weight data obtained.

**Results:**

Trip frequency was associated with gender, age, physical function, walking-aid use, educational attainment, number of amenities within walking distance and cars in the household. Participants reported 9.6 (SD 4.2) trips per week (trips·wk^-1^). Most trips (61%) were by car (driver 44%, passenger 17%), 30% walking or cycling (active) and 9% public transport/other. Driving trips·wk^-1 ^were more common in participants who were males (5.3 SD 3.6), well-educated (5.0 SD 4.3), high functioning (5.1 SD 4.6), younger (5.6 SD 4.9), affluent area residents (5.1 SD 4.2) and accessing > one car (7.2 SD 4.7). Active trips·wk^-1 ^were more frequent in participants who were males (3.4 SD 3.6), normal weight (3.2 SD 3.4), not requiring walking aids (3.5 SD 3.3), well-educated (3.7 SD 0.7), from less deprived neighbourhoods (3.9 SD 3.9) and with ≥ 8 amenities nearby (4.4 SD 3.8).

Public transport, and active trip frequency, were significantly associated with steps·d^-1 ^(p < 0.001), even after adjustment for other trip modes and potential confounders. Public transport, active, or car driving trips were independently associated with minutes MVPA·d^-1 ^(p < 0.01).

**Conclusions:**

Daily trips are associated with objectively-measured PA as indicated by daily MVPA and steps. Public transport and active trips are associated with greater PA than those by car, especially as a car passenger. Strategies encouraging increased trips, particularly active or public transport trips, in OAs can potentially increase their PA and benefit public health.

## Background

In the UK, the number of adults aged over 65 years increased between 1983 and 2008 by 1.5 m and those over 85 years increased from 600, 000 to 1.3 million [1]. Current projections suggest that those over 85 years will double in number by 2033. It is, therefore, increasingly important to find ways of facilitating the maintenance of physical function, health and independence and quality of life of older individuals. This in turn will help reduce the substantial financial and personal burden of health and social care costs incurred by the older adult population. Physically active older adults have lower risk of disease including dementia, higher levels of physical and cognitive function, psychosocial well-being and independence than inactive older adults [2]. However, less than 10% of those over 75 years meet the minimum amounts of activity recommended for health (30 minutes of at least moderate physical activity on five or more days per week) [3].

Both recreational physical activity (e.g., walking, gardening, bowls, exercise classes and swimming) and activity undertaken while performing daily tasks such as shopping and visiting friends (e.g., walking and cycling) are recommended for increasing overall levels of physical activity in older adults [4]. However, participation rates in recreational physical activity for those over 70 years is limited (walking 27.9%, swimming, 8.4%, keep fit and yoga 6.4%, bowls 4.8%, golf, 4.4% cycling 3.2%) [5]. National Travel Survey data [6] provide some indication as to the frequency and mode of transport for trips made from home. In adults over 70 years, 38% of all trips made were as a driver of a car, 23% as a passenger, 21% on foot, 12% by bus and just 1% by bicycle. The purposes of these trips are diverse with shopping accounting for 39%, sport and entertainment for 8%, and going for a walk (for leisure) 5% of all trips.

Although much research has been conducted on structured programmes of physical activity [7] much less is known about daily patterns of movement and their association with overall levels of physical activity [8]. Recent evidence from Japan indicates that getting out and about in the local neighbourhood is beneficial for maintaining physical function in the frail elderly [9].

Our own research with Project OPAL (Older People and Active Living) - http://www.bristol.ac.uk/enhs/opal - has also investigated these factors in older adults. Project OPAL was designed to provide comprehensive assessment of patterns and levels of activity, functionality, well being and perceptions of the environment. We have previously reported the associations between trips per week and of accelerometer assessed PA [10,11] as well as the association between neighbourhood deprivation and physical activity in 240 UK adults aged 70 and over. We found that trip frequency was one of a number of correlates of the daily steps (steps·d^-1^) and moderate to vigorous physical activity (MVPA) compared to those who made least (< 7) trips per week (p < .001) [10] and warranted further exploration. This study aims to describe the frequency, purpose, and travel mode of daily trips in adults over 70 years (y), and their association with participant characteristics and objectively assessed PA.

Understanding the nature of this relationship is important because it is currently unclear whether policy to increase activity in older adults should focus on the provision of facility-based structured exercise programmes or facilitation of free-living activities based in the local community.

## Methods

### Sampling and recruitment

A diverse sample of participants over 70 years were recruited to Project OPAL by written invitation via the patient lists of general medical practices distributed within the boundaries of a large city in the UK (Bristol). Practices were stratified by amenity access (the number of patients within each practice from areas with either low ≤0.38 k, or high ≥1.50 k, proximity to the nearest shop as defined by the English Index of Multiple Deprivation [IMD]). IMD combines 38 economic, social and housing indicators into a single deprivation score for each locality, with a high score denoting a high level of deprivation [12]. A three by two sampling matrix based on tertiles of IMD and the top and bottom 10% of amenity access was used to select 12 practices distributed across Bristol with a broad range of social economic groups and environmental settings.

Participants were randomly selected from patient lists and minimal exclusion criteria (namely: 1) recent bereavement, 2) terminal illness, 3) debilitating mental illness, 4) inability to complete a questionnaire, 5) any other illness preventing participation) were employed to maximise the diversity of the sample. Invitations to participate, an information pamphlet, and consent form were mailed to those patients who were not excluded by the practice administrator. Return of the consent form to the research team initiated inclusion in the project. The study was approved by the Bristol Southmead Research Ethics Committee (Reference 06/Q2002/127). Data was collected between April 2007 and December 2008

### Measures

Physical activity was assessed through accelerometry (Actigraph GT1Ms). Participants were supplied with an Actigraph and briefed on its use at the first (visit #1) of two home visits. Participants were asked to wear the Actigraph for seven days during waking hours, removing it only for bathing, water-based activities or when suffering discomfort. The instrument was worn in a custom Velcro™ pouch attached to the participant's own belt or a supplied elastic belt. Actigraphs were programmed to record activity in 10-second epochs, producing both count and pedometer data.

Also at visit #1 participants were supplied with and briefed on how to complete the daily trips log. The daily trips log was used to record details of the days and times when the Actigraph was worn and any trips made away from the home. For each trip, participants recorded the purpose (shopping, personal business [e.g., banking or posting letters], visiting friends or family, sport or exercise, day trip or excursion, going for a walk or walking the dog, escorting a friend or relative, work or volunteer activity, entertainment or going out to eat or drink, or "other") and in addition, the main mode of transport (walking, cycling, driving, car passenger, bus, train, or "other") for each trip was recorded.

Also during visit #1 height and weight were measured using stadiometer and portable scales respectively, and physical function was assessed using the Short Physical Performance Battery (SPPB) [13]. Demographic data were collected through an interviewer-administered questionnaire. Participants were asked to report their highest level of education completed (options were: primary school, middle school, some secondary school, completed secondary school, some college or vocational training, completed college or university, completed graduate degree or higher), these categories were later collapsed to three groups: primary/middle (includes those did some, but did not complete secondary school), secondary, and tertiary (some college or vocational training and above). Participants were asked how many drivable motor vehicles there were at the household and whether they regularly used a Zimmer frame, walking stick or other walking or mobility aid. The participant's residential postcode was used to derive the relevant Index of Multiple Deprivation (IMD) score. Further, participants were asked to indicate from a check list which amenities were perceived to be within a five-minute walk from their home. At visit #2 (usually seven to nine days after visit #1) the accelerometer and log were retrieved and responses to any remaining unanswered questions from the questionnaire recorded.

### Data reduction and analyses

Logs were inspected and entries for specified "other" trip purposes tabulated. Any specified options in the "other" category that were found to map onto existing options were re-coded to that option. Frequently occurring "other" options that did not map onto existing options were reclassified into new discrete options ("health" e.g., visit to hospital or GP, "religion" e.g., going to church, "gardening" e.g., tending an allotment or other remote garden, "hobby" e.g., playing musical instrument or card games away from home). Reclassification was performed by a researcher and decisions checked and confirmed by another researcher who was familiar with the data. The date of data collection was used to identify the current season and allow determination of seasonal influences on trips.

Actigraph data were downloaded using Actilife Lifestyle Monitoring System v. 3.1.3 software. Files failing to meet the inclusion criteria of ten hours of monitoring on at least five days, were excluded from analysis. Trip logs with fewer than five days of entries were also excluded. Both log data (number of trips) and accelerometry data were summed and then divided by the number of days for which data was collected (e.g., steps per day). For ease of interpretation a weekly equivalent trip frequency score was derived by multiplying the daily score by seven and this was used in analyses. Actigraph data were then reduced using MAH/UFFE Analyser v. 1.9.0.3 [14] set to ignore runs of 100 minutes of zeros. Prior investigation [15] has indicated that long periods of zero counts are not uncommon in this population and that setting this parameter any lower may risk distorting the data provided by the least active participants. Daily steps (steps·d^-1^) and minutes of at least moderate physical activity (≥1952 counts per minute, ≥ 3METs) (MVPA) were derived via batch processing.

Data were first checked for normality. Non-normally distributed data were transformed using the formula log [x+1]. Independent *t*-tests or one way analysis of variance (ANOVA) were used to determine differences between groups. Bivariate correlations were used to establish the strength of relationships between weekly trips and physical activity. The unadjusted association between respondent characteristics and trips per week separately for males and females was examined using one-way ANOVA. Each independent variable with a P value < 0.05 in the ANOVA was treated as a covariate in a series of ordinary least squares regression models to examine the association between the frequency of weekly trips by mode of travel, steps per day and MVPA. We have previously shown that gender is not associated with physical activity in this population. Therefore, for this reason and to retain power we did not run gender specific models.

## Results

### Participants

Of the 1172 older adults invited to participate, 662 were females (mean age 78.6 ± 8.6 years [y]) and 510 were males (mean age 77.5 ± 5.6 y). Responses were received from 725 individuals, 481 declined to participate, 244 gave informed consent to participate and 240 completed the study. The overall recruitment rate from those invited to take part was 20.8%. Although recruitment of members of this age group for physical activity studies is challenging [16] a representative sample for gender, age, and BMI was achieved. The age and gender of the sample differed minimally from the patient lists from which they were selected. The differences in proportions between pooled practice lists and recruits in each age group were: males 70-74 y -6.5%, 75-84 y +4.4%, 85-89 y +0.9%, ≥ 90 y +1.2%; females 70-74 y -4.4%, 75-84 y -7.0%, 85-89 y +7.8%, ≥90 y +3.6%. Participants' IMDs were fairly representative of the IMD distribution in England [17] (distribution within national tertiles: low, 30.4%, mid, 38.8, high 30.8%). From the 240 study participants, 16 participants failed to provide trip logs with at least five days of data, three failed to meet the inclusion criteria for accelerometer data (≥5 days of data), and seven failed to provide both valid accelerometer and valid log data. There were 214 participants who provided both accelerometry and log data that met the inclusion criteria.

### Trip frequency

The 214 participants recorded a total of 2007 trips over the seven days of recording. Only two participants did not perform any trips. Mean trips per week were 9.6 (SD 4.2), median trips per week were 9.0, and were normally distributed (Skewness 0.612, Kurtosis 0.215). The distribution among trip frequency categories was: low (< 6.0 trips·wk^-1^) n = 44, low-mid (6.0-8.9 trips·wk^-1^) n = 58, mid-high (9.0-12.9 trips·wk^-1^) n = 56, high (≥13 trips·wk^-1^) n = 56. Trip frequencies for selected participant characteristics are displayed in the last column of Table 1. Females recorded 1.8 fewer trips·wk^-1 ^than males (p = 0.008). Significantly fewer trips·wk^-1 ^were recorded by older participants, those low in physical function, using walking or mobility aids, educated to a lower level, living in more deprived areas, reporting fewer amenities within a five-minute walk of their home and living in households with just one car or no car at all. There were no significant trip frequency effects for BMI, living alone or season (F[3] = .450, p = .717).

**Table 1:** Mean trips per week made by different transport modes for selected participant characteristics

		**Trips·wk^-1 ^(mean ± SD) **^**a**^				
		**Transport mode**				
	**n**	**Drive (car)**	**Passenger (car)**	**Active**	**Public/other**	**Total**
**Gender**						
**Female**	105	2.6 ± 2.7	2.2 ± 2.5	2.6 ± 2.7	1.0 ± 1.8	8.7 ± 3.9
**Male**	109	5.3 ± 3.6	1.0 ± 1.5	3.4 ± 3.6	0.5 ± 1.2	10.5 ± 5.6
**T-test**		t = -4.7, p < .001	t = 4.4, p < .001	t = -1.8, p = .08	t = 2.6, p = .01	t = 2.7, p = .008
**Age group**						
**70-74.9 y**	76	5.6 ± 4.9	1.5 ± 2.2	3.6 ± 3.6	0.6 ± 1.3	11.5 ± 5.4
**75-79.9 y**	58	4.6 ± 4.2	1.5 ± 1.9	2.9 ± 3.1	0.6 ± 1.1	9.9 ± 4.7
**80-84.9 y**	54	2.4 ± 3.3	1.6 ± 2.0	2.6 ± 2.6	1.2 ± 2.1	8.2 ± 3.5
**≥85 y**	26	1.1 ± 1.9	2.0 ± 2.4	2.5 ± 3.2	0.6 ± 1.6	6.4 ± 4.2
**ANOVA**		F = 11.4, p < .001	F = 0.4, p = .725	F = 1.3, p = .280	F = 2.4, p = .068	F = 9.9, p < .001
**BMI category**						
**Normal (< 25 kg·m^2^) **^**b**^	70	3.6 ± 4.6	1.4 ± 2.2	3.2 ± 3.4	1.4 ± 2.1	9.9 ± 4.6
**Overweight (25.0-29.9 kg·m^2^)**	86	4.2 ± 3.7	1.4 ± 1.8	3.5 ± 3.4	0.3 ± 0.8	9.6 ± 5.2
**Obese (≥ 30 kg·m^2^)**	58	4.1 ± 4.9	2.0 ± 2.4	2.0 ± 2.4	0.6 ± 1.3	9.2 ± 4.8
**ANOVA**		F = 0.4, p = .701	F = 2.1, p = .120	F = 3.9, p = .022	F = 10.4, p < .001	F = 0.4, p = .699
**Physical Function (SPPB score category)**						
**Low (≤6)**	28	1.0 ± 1.4	2.1 ± 2.4	1.7 ± 2.8	0.5 ± 1.4	5.4 ± 3.6
**Mid (7-9)**	50	2.5 ± 3.2	1.6 ± 1.8	2.1 ± 2.3	0.9 ± 1.7	7.6 ± 3.8
**High (≥10)**	136	5.1 ± 4.6	1.5 ± 2.1	3.6 ± 3.4	0.8 ± 1.5	11.2 ± 4.9
**ANOVA**		F = 15.6, p < .001	F = 0.9, p = .401	F = 7.1, p = .001	F = 0.5, p = .619	F = 26.9, p < .001
**Walking and mobility aid use**						
**None**	153	4.6 ± 4.4	1.5 ± 2.1	3.5 ± 3.3	0.7 ± 1.5	10.6 ± 4.7
**Walking aid**	55	2.4 ± 3.7	1.8 ± 2.0	2.6 ± 2.6	0.9 ± 1.8	7.5 ± 4.7
**Mobility aid**	6	2.2 ± 3.4	2.5 ± 2.3	0.0	0.3 ± 0.5	5.2 ± 2.1
**ANOVA**		F = 6.1, p = .003	F = 1.0, p = .368	F = 7.0, p = .001	F = 0.4, p = .664	F = 11.8, p < .001
**Education **^**c**^						
**Primary/middle**	44	2.3 ± 4.1	1.9 ± 2.1	1.9 ± 2.3	0.8 ± 1.6	7.4 ± 4.7
**Secondary**	65	3.5 ± 4.1	1.7 ± 2.3	2.6 ± 2.4	1.0 ± 2.0	9.2 ± 4.6
**Tertiary**	104	5.0 ± 4.3	1.3 ± 1.9	3.7 ± 0.7	0.6 ± 1.2	10.9 ± 4.8
**ANOVA**		F = 6.7, p = .002	F = 1.3, p = .274	F = 6.1, p = .003	F = 1.4, p = .251	F = 9.1, p < .001
**IMD **^**d**^						
**Low**	70	5.1 ± 4.2	1.5 ± 2.2	3.9 ± 3.9	0.5 ± 1.1	11.3 ± 4.7
**Mid**	73	3.4 ± 4.0	1.7 ± 2.3	2.3 ± 2.6	0.9 ± 1.8	8.6 ± 4.5
**High**	71	3.5 ± 4.7	1.5 ± 1.9	2.8 ± 2.8	1.0 ± 1.7	8.9 ± 5.2
**ANOVA**		F = 3.3, p = .038	F = 0.2, p = .801	F = 4.7, p = .010	F = 2.1, p = .121	F = 6.5, p = .002
**Amenities within 5-min walk category **^**e**^						
**None**	18	1.3 ± 2.4	2.2 ± 2.7	1.4 ± 2.2	0.6 ± 1.5	5.8 ± 4.0
**1**	26	3.3 ± 3.9	2.1 ± 2.0	2.7 ± 3.3	0.5 ± 0.8	8.7 ± 4.3
**2-3**	56	4.4 ± 4.6	1.7 ± 2.0	2.6 ± 2.7	0.6 ± 1.4	9.7 ± 4.6
**4-7**	59	4.5 ± 4.6	1.1 ± 1.9	2.6 ± 2.9	1.0 ± 1.9	9.8 ± 5.1
**≥8**	55	4.2 ± 4.2	1.5 ± 2.2	4.4 ± 3.8	0.8 ± 1.6	11.1 ± 4.9
		F = 2.3, p = .064	F = 1.6, p = .178	F = 4.5, p = .002	F = 0.8, p = .508	F = 4.5, p = .002
**Home circumstances **^**f**^						
**Live alone**	77	3.3 ± 4.2	1.1 ± 1.7	3.0 ± 2.7	1.3 ± 2.0	9.0 ± 4.5
**Live with others**	128	4.4 ± 4.4	1.9 ± 2.3	3.0 ± 3.5	0.5 ± 1.2	10.0 ± 5.2
**T-test**		t = 1.8, p = .079	t = 2.8, p = .008	t = 0.1, p = .885	t = -3.7, p = .001	t = 1.4, p = .158
**Number of cars in household**						
**None**	52	0.3 ± 0.9	1.4 ± 1.5	2.8 ± 2.7	1.9 ± 2.3	6.7 ± 3.2
**1**	126	4.7 ± 4.1	1.5 ± 2.2	3.2 ± 3.4	0.4 ± 1.0	10.1 ± 4.9
**≥2**	34	7.2 ± 4.7	2.1 ± 2.3	2.6 ± 3.4	0.3 ± 0.6	12.4 ± 4.9
**ANOVA**		F = 41.6, p < .001	F = 1.3, p = .276	F = 0.7, p = .505	F = 24.0, p < .001	F = 17.5, p < .001

^a ^Trips per week calculated by number of trips made divided by the number of days for which log data was provided that could be matched with accelerometer data, multiplied by 7. ^b ^Normal weight includes eight participants whose BMI was < 18.5 kg·m2 (range 16.0 - 18.33 kg·m^2^). **^c ^**Highest level of education achieved. Primary/middle includes those did some, but did not complete secondary school. Tertiary includes college and university education. One participant did not provide information on education. ^d ^IMD, Index of Multiple Deprivation. **^e ^**Amenities within 5-min walk category, number of amenities (e.g., shop, post office, park) that were perceived to be within a 5-minute walk of the participants home. ^f ^Seven females and two males failed to provide data on home circumstances. See Figure 2 for notes on transport modes.

### Purposes of trips

**Figure 1:**
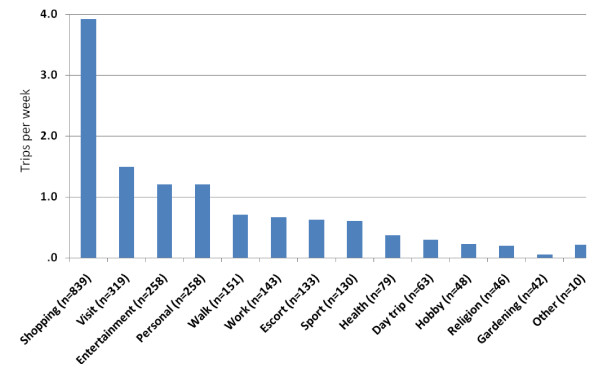
**Mean trips per week for different trip purposes**. Mean trips per week for all 214 participants regardless of whether they did any trips for these purposes. Multiple purposes allowed for a single trip. Shopping: shopping but not post office or banking activities that would be covered by personal business. Visit: visiting friends and family. Entertainment: activities such outings for a meal, drink, cinema and any other entertainment activities including watching sport. Personal: personal business activities such as post office business, banking dry cleaning. Walk: going for a walk or walking the dog. Work: doing paid work or volunteer activities. Escort: taking or accompanying a friend or relative (e.g., grandchild) to another venue (e.g., school). Sport: active involvement in a sporting activity or exercise session. Health: visiting a health practitioner (e.g., GP, dentist), hospital, clinic, or pharmacy. Day trip: going on a day trip, excursion or short break. Hobby: attending arts or crafts activity groups or games groups (e.g., playing cards). Religion: attending a religious service or meeting. Gardening: doing gardening or allotment work away from the confines of the participant's home (not attending to own garden). Other: unspecified activities or activities not covered by the previous categories.

**Figure 2:**
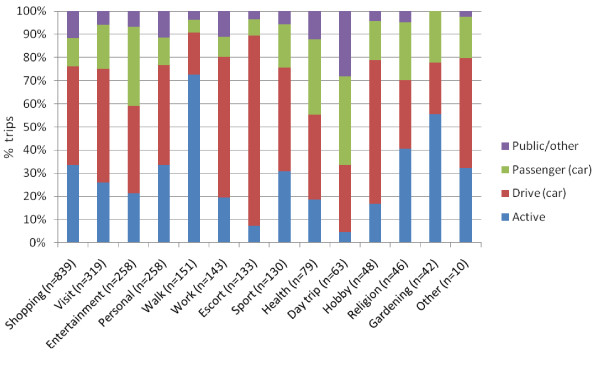
**Transport modal split for different trip purposes**. Public/other: trips made by bus, train or unspecified other transport. Passenger (car): trips made as passenger in a car. Drive (car): trips made as a driver of a car. Active: trips made on foot or by bicycle. See figure 1 for key to purposes.

Trip purposes (see Figure 1) were shopping (33.2%), visiting friends or family (12.7%), entertainment (10.2%), personal business (10.2%), going for a walk (6.0%), work or volunteer activities (5.7%), escorting a friend or relative (5.3%), sport or exercise (5.2%), visiting a GP or other health-related visit (3.1%), going on a day trip (2.5%), hobby (1.9%), religion or church attendance (1.8%), allotment or gardening (1.7%), other (0.4%) (N.B. multiple purposes for a single trip were allowed the proportions presented are for all purposes [n = 2519] as opposed to trips). The mean trip frequency per week by trip purposes is presented in Figure 2. The most frequent purpose for a trip was for shopping (3.9 SD 2.7 trips·wk^-1^). Other trip purposes that occurred at least once per week were visiting others (1.5 SD 1.5 trips·wk^-1^), entertainment, e.g., going out for a drink or a meal (1.2 SD 1.5 trips·wk^-1^) and personal business (1.2 SD 1.4 trips·wk^-1^). Males reported more shopping trips per week (5.0 SD 3.1 trips·wk^-1^) than females (3.5 SD 1.8, p < .001) with the difference largely accounted for by the higher overall trip frequency recorded by males.

### Modes of transport used for trips

Trips (including those for the purpose of just going for a walk) were made by car (driving 42.7%, passenger 16.8%), through physical activity (walking 31.2%, cycling 1.1%) by public transport or other means (bus 7.2%, train 0.4%, other 0.7%). The proportion of trips made by these different modes of transport is shown by trip purpose in Figure 2. The proportion of active trips was greater than car-based trips for walking, gardening and religion but these accounted for just 6.0% of all trip purposes. For most purposes of trip, and for the most frequently reported purposes of trip (shopping), the car was the most frequently used mode of travel.

Males made twice as many trips·wk^-1 ^as a car driver than females, whereas females made twice as many trips·wk^-1 ^as a passenger or by public transport (see Table 1). Trips·wk^-1 ^as a car driver declined with age and physical function. Those 70.0-74.9 y and high in physical function made five times as many trips·wk^-1 ^as a car driver as those ≥ 85 y or low in physical function respectively. Those who did not use a walking or mobility aid made twice as many trips as a car driver than those who used such aids. These differences in trip frequency for car driving were not compensated for by other travel modes.

### Association of trips with physical activity

There was a consistent moderate correlation for the frequency of trips with both daily steps (range R .367 - .505, p < .001) and MVPA (range R .361 - .472, p < .001) across the different days of the week (see Table 2). There was also a correspondingly lower level of activity on weekend days when there were fewer trips made. Table 3 shows the association between trips·wk^-1 ^by mode of travel, steps ·d^-1 ^and MVPA·d^-1^. Age and sex adjusted trips per week accounted for 46% of the variance in steps ·d^-1 ^(adjusted R^2 ^0.46) and 42% of the variance in MVPA·d^-1 ^(adjusted R^2 ^0.42). Each weekly trip made by public transport is associated with an additional 478 steps ·d^-1 ^(SE 93.8, p < 0.001). Corresponding values for car trips as a driver and walking/cycling trips are 166 steps ·d^-1 ^(SE 36.5, p < 0.001) and 352 steps ·d^-1 ^(SE 40.3, p < 0.001). Trips as a car passenger were not associated with steps ·d^-1^. Following mutual adjustment for other trip types, age, sex, physical function, use of a walking aid, education and car ownership, the association between trips taken by public transport and walking or cycling were attenuated somewhat but remained significant. Car driver trips were no longer associated with steps ·d^-1^. Trips by public transport, car driving and walking/cycling were also associated with MVPA·d^-1 ^(p < 0.01) even after adjustment.

**Table 2:** Weekly patterns of trips and associations with corresponding daily steps and minutes of MVPA

		**Trips·d**^**-1**^	**Steps·d **^**-1**^	Association of steps with trips		**Min. MVPA·d **^**-1**^	Association of MVPA with trips	
	**N**	**Mean ± SD**	**Mean ± SD**	**R**	**p**	**Mean ± SD**	**R**	**p**
**Monday**	209	1.3 ± 1.0	4704.8 ± 3150.4	.445**	< .001	19.6 ± 23.2	.420**	< .001
**Tuesday**	205	1.5 ± 1.0	4587.4 ± 2944.2	.412**	< .001	18.3 ± 20.9	.377**	< .001
**Wednesday**	206	1.5 ± 1.0	4721.2 ± 2986.0	.455**	< .001	19.2 ± 22.4	.316**	< .001
**Thursday**	208	1.5 ± 1.0	4582.2 ± 2860.5	.464**	< .001	19.0 ± 20.9	.395**	< .001
**Friday**	211	1.4 ± 1.0	4738.0 ± 2943.8	.444**	< .001	19.8 ± 22.6	.366**	< .001
**Saturday**	209	1.2 ± 1.0	4296.9 ± 2774.0	.505**	< .001	17.0 ± 20.1	.472**	< .001
**Sunday**	208	.9 ±.8	3541.8 ± 2857.3	.367**	< .001	14.3 ± 22.4	.368**	< .001

Min. MVPA, minutes of at least moderate physical activity (> 1951 CPM).

**Table 3:** Regression examining the association between trips·wk-1 by different transport modes with steps·d^-1 ^and minutes of MVPA·d^-1^

	**Steps·d**^**-1**^				Min. MVPA·d^-1^(ln)			
	**Model 1**		**Model 2**		**Model 1**		**Model 2**	
	**b**	**(SE)**	**b**	**(SE)**	**b**	**(SE)**	**b**	**(SE)**
**Public/other**	478.1***	-93.8	412.7***	-87.5	0.09***	0	0.06***	0
**Passenger (car)**	92.43	-69.4	10.35	-66.7	0.02	0	0.01	0
**Drive (car)**	166.3***	-36.5	70.99	-37.6	0.03***	0	0.02**	0
**Active**	352.3***	-40.3	285.0***	-38.6	0.05***	0	0.03***	0
**Adjusted R**^**2**^	0.46		0.56		0.42		0.6	

Steps·d^-1^, steps per day. Min. MVPA·d^-1^, minutes of moderate to vigorous physical activity per day. Ln, log transformed data. Model 1: Adjusted for age, sex and mutual adjustment for other modes of travel; Model 2: Model 1 plus adjustment for physical function, use of a walking aid, education and car ownership; **p < 0.01, ***p < 0.001; SE: Standard error.

## Discussion

This study aimed to assess the relationship between frequency, purposes and transport mode of daily trips, with participant characteristics and with accelerometry-assessed daily physical activity. We have combined objective data from a diverse sample of older adults on physical activity, physical function and self report data on trips made out of the home (including frequency, purposes and modes of travel for trips). We believe this study is unique in this respect. The associations we have found for trips and their modes and purposes with physical activity and with participant characteristics help improve our understanding of the importance of "getting out and about" behaviour for older adults.

Levels of PA and MVPA in older adults are low [3], and were so in this sample [10], so it is important to identify lifestyle and demographic factors that contribute to both PA and MVPA. We found that the frequency of making trips away from the home is associated both with increased walking and time spent in MVPA on a daily and weekly basis. We also found that weekend days produced least trips occurred and this coincided with lowest daily levels of PA. This reconfirms the important contribution that daily trips make to PA in older adults.

Active trips, and those by public transport make bigger contributions to PA than by car. Even after adjustment for potential confounders a trip outdoors each day by foot or bicycle is associated with an estimated extra 20 minutes of daily walking (assuming 100 steps per minute [18]) and 13 minutes of MVPA. Equivalent values for a daily trip by public transport are 29 minutes of daily walking and 20 minutes of MVPA. These results confirm previous research [19] showing use of public transport was associated with higher physical activity when compared to private motorised transport. Our findings also suggest that not only does public transport offer increased opportunities for getting out and about but may provide additional important contributions to physical activity over and above that generated by getting out of the house. Not only was there an association for public transport with the volume of walking, but also there was an association with the intensity of activity (MVPA). This is an important effect in a population where volumes of MVPA are low and suggests that the walking component of trips made by public transport may be made at a brisk pace.

Although active travel and use of public transport are associated with higher levels of activity, there is a very strong reliance by older people on use of the car for journeys away from home. Males tended to drive and women were passengers. This is supportive of National Transport Survey data [6] providing similar findings (driving trips: males 57%, females 21%, passenger trips males 9%). Being a frequent driver is associated with higher activity but being a passenger is not. Possible explanations are that car passengers have lower function, and are more likely to report using a walking aid than car drivers. Alternatively it may be that passengers are not actively involved in the trip purpose (i.e., they stay in the car).

While available, the car probably assists in helping older adults make more trips, with lower life expectancies in males [20], this poses a potential challenge for widowers previously reliant on their spouse as the main driver. Therefore older women are more dependent on trips that can be made on foot or by public transport which in turn requires personally important destinations that can be reached by these modes of travel.

While there was a wide variety of purposes of trips for older adults, shopping accounted for a third of all trips, similar to that found in the NTS that reported 39% of trips were for shopping [6]. The NTS showed that the proportion of trips for commuting and business decline steeply after age 59 y as the proportion of trips for shopping, personal business and visiting friends and family increase. So it appears that shopping is the main reason for making a trip once people retire from work. Relatively few trips are made for leisure and fitness purposes, including going for a walk for pleasure only or to take part in sport or exercise.

Access to shops and other services would therefore seem important for encouraging trips out. We found that having several amenities within five minutes' walk of home was associated with more frequent trips across all transport modes combined. However, the effect was particularly prominent for trips on foot or by bike. If we are to promote increased levels of walking and public transport trips it follows that these type of destinations need to be accessible via these modes of travel. The alternative is further reliance on the car and the problem it brings in terms of congestion, pollution and its loss when driving is no longer possible.

### Limitations

Our data are cross-sectional and as a result we cannot determine the direction of the relationship between frequency, purpose and mode of trips with physical activity. It is possible that physically active people take more trips rather than trips leading to more physical activity. Therefore longitudinal data are needed to confirm or refute our findings. The trip data is by daily recall and there may be some misclassification of trip frequency. However, as long as any misclassification was not differential with regards to physical activity then the effect is likely to attenuate rather than exaggerate any associations between trip frequency and physical activity.

We aggregated trip data to weekly totals and averaged the weekly volume of physical activity to daily totals and show a general association between trip frequency and physical activity. This may reduce the heterogeneity of the data and weaken the argument about the temporal specificity of the relationship. A multilevel analysis with days clustered within individuals may have added precision to the association by directly linking trips on a given day to physical activity on the same day. The study was not designed to accommodate a multilevel analysis but this would be of value in future studies. Nevertheless we provide a close examination of associations between trips and PA on separate days of the week and have found consistent relationships across the week suggesting that trips are associated with PA on the same day rather than trips simply being indicative of a more active lifestyle.

There is debate over whether MVPA cut points used for younger adults are appropriate for older adults and those with impaired mobility [21]. Until it is practical to compute relative measures of intensity this issue is recognised as a limitation of accelerometer-based studies of older adults.

## Conclusions

Trips away from the home are associated with objectively measured physical activity, both as volume of MVPA, and steps per day. Shopping is an important activity, and combined with trips for personal business accounted for nearly half of all trips out of the house. Increasing opportunities and convenience for shopping and undertaking personal business for older adults should support more physical activity. However, the mode of transport used for trips away from the home is also important. Trips made by public transport, or walking provide more activity than those made by car, and especially as a passenger in a car. Strategies to encourage increased trips in older adults, particularly those by active means or via public transport have the potential to increase physical activity in older adults and benefit public health.

Therefore, local shops and services that are within walking distance or accessible through public transport should be regarded as an important local resource for enhancing physical activity in older adults.

## Abbreviations

ANOVA: analysis of variance; BMI: body mass index; GP: general practitioner; IMD: Index of Multiple Deprivation; Ln: log transformed; METs: metabolic units: MVPA·d^-1^: moderate to vigorous physical activity per day; NTS: National Travel Survey; OPAL: Older People and Active Living; PA: physical activity; PF: physical function; SD: standard deviation; SE: standard error; SPPB: Short Physical Performance Battery; Steps·d^-1^: steps per day; Trips·wk^-1^: trips per week; Y: years.

## Competing interests

The authors declare that they have no competing interests.

## Authors' contributions

MD conceived and designed the study of trips and association with objectively measured physical activity. KF was primary investigator for Project OPAL, from which the data was derived. KF, MH and JT contributed the design of the study, analysis of data and interpretation of results. JC was involved in data acquisition. DS contributed to design of recruitment strategies. KF, MH, JT, DS and AS were co-applicants for Project OPAL and contributed to its conception and design. All authors read and approved the final manuscript.
